# Ptch1 and Gli regulate Shh signalling dynamics via multiple mechanisms

**DOI:** 10.1038/ncomms7709

**Published:** 2015-04-02

**Authors:** Michael Cohen, Anna Kicheva, Ana Ribeiro, Robert Blassberg, Karen M. Page, Chris P. Barnes, James Briscoe

**Affiliations:** 1 MRC-National Institute for Medical Research, The Ridgeway, Mill Hill, London NW7 1AA, UK; 2 Department of Mathematics and CoMPLEX, University College London, Gower Street, London WC1E 6BT, UK; 3 Department of Cell and Developmental Biology, University College London, Gower Street, London WC1E 6BT, UK; 4 Department of Genetics, Evolution and Environment, University College London, Gower Street, London WC1E 6BT, UK

## Abstract

In the vertebrate neural tube, the morphogen Sonic Hedgehog (Shh) establishes a characteristic pattern of gene expression. Here we quantify the Shh gradient in the developing mouse neural tube and show that while the amplitude of the gradient increases over time, the activity of the pathway transcriptional effectors, Gli proteins, initially increases but later decreases. Computational analysis of the pathway suggests three mechanisms that could contribute to this adaptation: transcriptional upregulation of the inhibitory receptor Ptch1, transcriptional downregulation of Gli and the differential stability of active and inactive Gli isoforms. Consistent with this, Gli2 protein expression is downregulated during neural tube patterning and adaptation continues when the pathway is stimulated downstream of Ptch1. Moreover, the Shh-induced upregulation of Gli2 transcription prevents Gli activity levels from adapting in a different cell type, NIH3T3 fibroblasts, despite the upregulation of Ptch1. Multiple mechanisms therefore contribute to the intracellular dynamics of Shh signalling, resulting in different signalling dynamics in different cell types.

## Abstract (web summary)

Gradients of the secreted morphogen Sonic Hedgehog (Shh) pattern the neural tube in vertebrates. Cohen *et al.* quantify Shh signalling in developing mice, and by constructing a computational model of the process, identify mechanisms by which the dynamics of Shh signalling are regulated.

## Graphical abstract

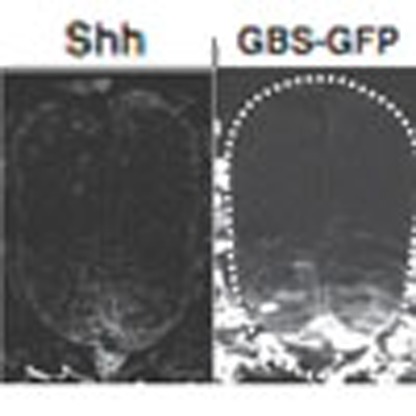

In several developing tissues, Sonic Hedgehog (Shh) acts as a morphogen, providing positional information to control cell fate decisions and organize the pattern of differentiation^1^,^2^,^3^. The same pathway is also important in adults for the regulation of cell proliferation and tissue homeostasis and aberrant signalling has been implicated in carcinogenesis^4^. Many of the components of the Shh transduction pathway have been identified (Fig. 1a); however, how the pathway produces the intracellular dynamics of signalling is less clear. In this study, we make use of experimental data and Bayesian computational techniques to interpret key features of the Shh signalling dynamics and infer details of the underlying molecular mechanisms.

Shh signalling is initiated when the secreted ligand binds to the transmembrane receptor Patched (Ptch1)^4^,^5^. Unliganded Ptch1 inhibits the activity of a second transmembrane protein, Smoothened (Smo), which in turn controls the downstream modification of Gli transcriptional effectors (Fig. 1a). Of the three Gli proteins in mammals, Gli2 acts predominantly as an activator and Gli3 as a repressor^4^,^5^. Gli1 is not expressed in the absence of signalling but is transcriptionally induced by Shh signalling and acts as an activator. In the absence of Shh, Smo is inactive and the full-length forms of Gli2 and Gli3 (GliFL) are proteolytically processed into repressive forms (GliR). The binding of Shh to Ptch1 activates Smo, which in turn leads to the inhibition of GliFL processing and the production of the transcriptionally active forms of Gli (GliA)^6^. The net Gli activity (which we shall refer to as the ‘signal’) that results from the balance between GliA and GliR regulates the expression of a number of target genes, including the receptor Ptch1, Gli1 (refs 7, 8) and transcription factors specifying neuronal identity^9^,^10^,^11^.

In the neural tube, Shh is secreted from the ventrally located notochord and floor plate and forms a concentration gradient along the ventral–dorsal axis of the neural tube^12^,^13^. The Shh gradient determines the boundary positions between molecularly distinct neural progenitor domains that generate different neuronal subtypes^2^,^11^. At least during the earliest stages of patterning, the amplitude of the Shh gradient increases at the same time as pattern is elaborated^13^. Quantitative analysis of downstream Gli activity indicates that the response of cells is also dynamic^10^,^14^,^15^. Initially Gli activity rapidly increases, reaching peak levels early in development (~E9 in mouse), and subsequently decreases to lower levels over time^10^,^14^,^15^. We refer to this temporal profile in net Gli activity level as adaptation^16^. The adaptation dynamics are crucial for the spatiotemporal profile of expression of Shh target genes, because the transcriptional network that connects them in neural progenitors responds to both the level and duration of Shh signalling^14^,^17^,^18^.

Similar adapting (or ‘pulse like’) signal responses have been identified in other signalling systems, for instance chemotaxis^19^,^20^, JAK2/STAT5 signalling^21^, Wnt signalling^22^, epidermal growth factor signalling^23^, transforming growth factor-β signalling^24^, calcium homeostasis^25^ and yeast osmoregulation. Various underlying mechanisms including negative feedback loops, incoherent feed forward loops and integral control have been described to produce these dynamics^26^,^27^. In each case, adaptation occurs by the inactivation or degradation of one or more of the pathway components—either at the level of transcription or by post-translational regulation.

In the neural tube, the transcriptional upregulation of the receptor Ptch1 in response to Gli activation contributes to the observed adaptation of Gli activity^16^,^17^. Because Ptch1 inhibits the activity of Smo, its transcriptional activation is suggested to reduce the levels of active Gli. While such negative feedback via receptor number seems an intuitively plausible mechanism for adaptation, it is complicated by the observation that the amplitude of the Shh gradient increases over time, hence cells are likely to experience an increasing ligand concentration^13^. Given that inhibition of the pathway relies on the availability of receptors that are unbound by Shh and can inhibit Smo, increasing amounts of Ptch1 might be required to absorb increasing amounts of Shh. However, Ptch1 transcript and protein levels also undergo adaption^14^,^18^,^15^. Previous analysis of negative feedback circuits of this kind has suggested that they may not adapt, but saturate, when stimulated by high input signals^27^ and are prone to oscillations^27^,^28^,^29^. This raised the possibility that other features of the pathway might contribute to the observed dynamics.

Biochemical experiments have indicated that the activation of Gli proteins by stimulation of the Shh pathway reduces their stability^30^,^31^,^32^,^33^,^34^. In addition, the genetic removal of SuFu, an inhibitor of Gli activation, results in the destabilization of Gli proteins^35^. Furthermore, the E3 ubiquitin ligase SPOP has been shown to compete with SuFu and also promote degradation of activated Gli^34^. Theoretical and experimental studies have demonstrated that adaptation through integral feedback may occur in systems where a protein is converted from a stable to an unstable form^19^,^36^,^37^,^38^,^39^,^40^. Whether the conversion by Shh of Gli proteins to a less stable active forms could account for the observed adaptation has not been explored.

Alternatively, or in addition, adaptation in signalling levels could rely on the transcriptional downregulation of the Gli genes and other components of the signalling pathway. Decreased production of critical components of the pathway would lower the overall levels of signalling and thus the levels of Gli activity. Indeed, the downregulation of Gli2 and Gli3 expression at late developmental stages has been observed in the neural tube^7^,^10^,^15^,^18^,^41^. However, Gli activity in the neural tube continues to adapt in the absence of Gli3 (ref. 14). Whether the spatiotemporal regulation of Gli2 transcription can explain the observed dynamics is unclear.

By contrast to neural progenitors, cultured embryonic NIH3T3 fibroblasts, which are routinely used as a model system for Shh signalling, do not appear to show adaptation in response to Shh^42^. Nevertheless, the components and overall structure of the signalling pathway appear to be qualitatively similar between these two cell types. Thus, any mechanistic model of the signalling pathway needs to explain how the same signalling pathway is refashioned to generate quantitatively different outputs.

Here we used a combination of experiment and computational modelling to establish a model of Shh signal transduction. We took advantage of a computational technique, Approximate Bayesian Computation (ABC)^43^ to obtain model parameter distributions through the fitting of dynamical simulations to observed experimental data. Using this method, we found that mechanisms involving Ptch1 transcriptional upregulation, the regulation of Gli isoform stability and Gli transcriptional downregulation, all have the potential to account for the adaptive dynamics in the neural tube. This was supported by experimental tests. Notably, the inspection of Gli2 transcription revealed that it is downregulated during neural tube patterning providing support for this mechanism of adaptation. By comparison, the sustained signalling observed in NIH3T3 fibroblasts could be accounted for by the upregulation of Gli2 transcription. Together these data provide insight into the mechanisms of distinct Shh signalling dynamics in different cell types.

## Results

### Dynamics of Shh gradient and signalling in the neural tube

**Figure 1:**
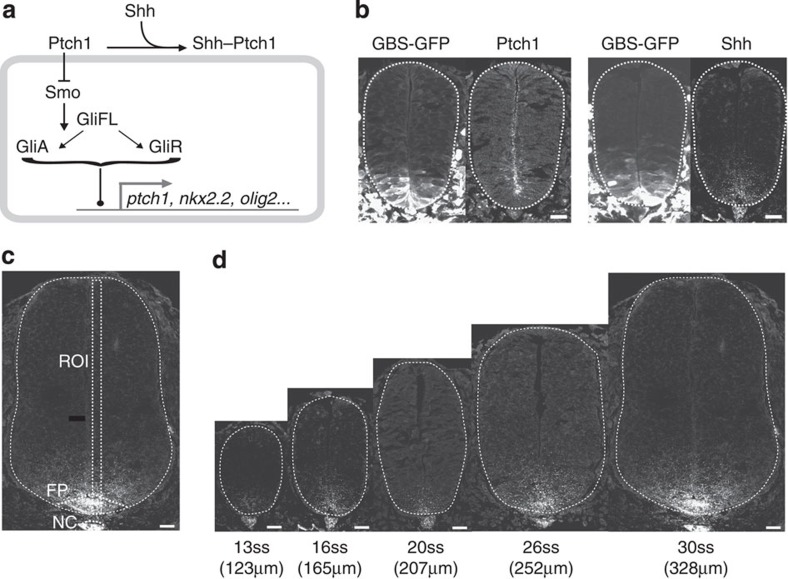
Shh levels increase in the neural tube. (**a**) Cartoon of the Shh pathway. In the absence of Shh, Ptch1 inhibits Smo. GliFL is processed into its repressive form GliR, which switches off the expression of target genes. In the presence of Shh, Ptch1 receptors bind to the ligand, derepressing Smo and converting GliFL to its active form, GliA, which promotes the target gene expression including ptch1. (**b**) Sections from embryos were immunostained for either GBS-GFP, Ptch1 and Nkx2.2 or GBS-GFP, Shh and Nkx2.2 (Nkx2.2 data not shown). Two sections from the same embryo at the 16-somite stage. Scale bar, 20 μm. GBS-GFP and Ptch1 data has been reproduced from ref. 14. (**c**) A neural tube section at the 30-somite stage immunostained for Shh. The mean Shh fluorescence intensity across a 16-μm wide region of interest (ROI) extending from ventral to dorsal midline, in steps of 1 μm, was quantified next to the apical lumen. The floor plate (FP) and notochord (NC) provide the source of Shh. Scale bar, 20 μm. (**d**) Shh immunostaining in embryos of the indicated somite stages (ss) from E8.5 (~10ss) to E10.5 (~40ss) Scale bar, 20 μm. The number in brackets represents the dorsal–ventral size of the neural tube.

While the long-range action of Shh is well-documented^44^,^45^, there is limited knowledge of the formation and temporal dynamics of the Shh gradient in the neural tube. Previous reports have shown that in mouse, Shh immunoreactivity close to the ventral midline is higher at E10.5 than E8.5, and that the levels of a Shh-GFP (green fluorescent protein) fusion protein increase during early development before E9.5 (refs 12, 13). Meanwhile, the downstream Gli activity in the target tissue peaks at E9 and subsequently undergoes temporal adaptation^14^,^15^. However, the precise relationship between Shh concentration and signalling dynamics in the neural tube had not been measured. To do this, we collected transverse brachial sections from 68 embryos expressing a transcriptional reporter of Shh signalling activity, Tg(GBS-GFP)^14^, at different time points from days E8.5 to E10.5 of mouse development and immunostained them for GFP, Shh and Ptch1 (Methods, Fig. 1b–d; ref. 14). Individual sections were staged by the dorsal–ventral length of the neural tube, which correlates tightly with the somite stage of the embryos (Methods, Supplementary Fig. 1a). All sections were immunostained and imaged in the same experiment, using the same conditions, and the fluorescence intensity was measured along the dorsal–ventral (D–V) axis of the neural tube in a 16-μm region adjacent to the apical lumen of the neuroepithelium (Fig. 1b–d, Methods).

**Figure 2:**
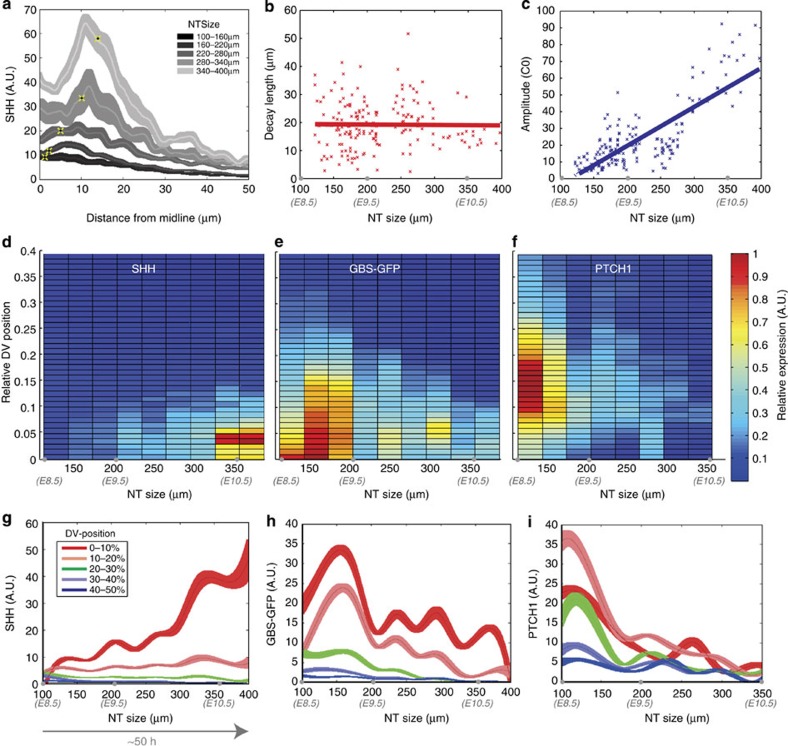
Quantitation of Shh signalling levels. (**a**) Mean Shh intensity for the indicated developmental stages along the dorsal–ventral axis. The width of the curves shows the 95% confidence interval for the mean. Yellow crosses indicate the mean position of the Nkx2.2 ventral boundary (s.e. <1.1 μm, not shown). The shift in the position of the maximum intensity over time is attributed to the changes in the size and position of the floor plate. (**b**,**c**) The decay lengths (**b**) and amplitudes (**c**) with linear trendlines from exponential functions fit to the Shh fluorescence intensity profiles. The average correlation coefficient for the exponential fits using Matlab’s non-linear least-squares method was *R*^2^=0.74. For the decay length, there was no correlation with neural tube tissue size, for the amplitude the correlation coefficient, *R*^2^=0.8 (*P*<0.0001). (**d**–**f**) Heat maps of mean Shh, GBS-GFP and Ptch1 fluorescence intensity (colour-coded) profiles. Embryonic stage on the *x* axis and distance from the ventral midline, relative to the total neural tube length, on the *y* axis. All profiles normalized to maximum intensity. Each panel represents data from 68 embryos, with at least three embryos per stage (Shh (*n*=185), GBS-GFP (*n*=206), Ptch1 (*n*=102)). The GBS-GFP and Ptch1 data are reanalysed data from ref. 14. (**g**–**i**) The data in **d**–**f**, plotted for several DV position ranges. On the *x* axis are different time points. The data were binned along the *x* axis with bin size of 40 μm and the curves interpolated by fitting a spline at 0.1 μm intervals. The curve width indicates 95% confidence intervals for the mean values.

Quantitation of the Shh fluorescence intensity profiles (Fig. 2a) revealed that the maximum intensity occurred between 5 and 13 μm away from the ventral midline. There was a good correlation between the position of the peak Shh intensity and position of the ventral boundary of the Nkx2.2 expression domain (indicated by green crosses in Fig. 2a)—a marker for the p3 progenitor domain, which after E9.5 abuts the more ventrally located Shh-producing floor plate cells (Supplementary Fig. 1b)^18^. This is consistent with previous observations that Shh protein is secreted and enriched on the apical surface of floor plate cells and its levels increase over time^13^. We therefore defined the position of the maximum Shh intensity as the boundary position between Shh source and target tissue. This allowed us to fit an exponential function 
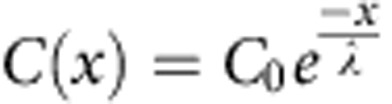
, where C is fluorescence intensity, *x* distance from the source, to the data. The gradient amplitude *C*_0_ and the exponential decay length *λ* were derived from the fit (Fig. 2b,c). This revealed that the decay length is variable between embryos, but on average, remains approximately constant regardless of the total size of neural tube (19.6±4.2 μm). In contrast, the amplitude *C*_0_ increased more than 10-fold over time and correlated linearly with tissue size (*R*^2^=0.8).

We compared the Shh profiles with the levels of intracellular signalling measured using Gli-binding sites (GBS)-GFP and Ptch1 immunostaining in the same set of embryos (Fig. 2d–i, Methods) (GBS-GFP and Ptch1 data is reproduced from ref. 14). Both GBS-GFP activity and Ptch1 protein formed ventral to dorsal gradients. By contrast to increasing levels of Shh over time (Fig. 2d,g), the GBS-GFP (Fig. 2e,h) Ptch1 protein (Fig. 2f,i), *ptch1* and *gli1* mRNA levels^14^,^10^,^15^ adapted over time. (An independent data set comprising 65 sections taken from 23 additional embryos revealed the same temporal adaptation in the GBS-GFP signal (Supplementary Fig. 1c; ref. 14).

### Evidence that Ptch1 feedback is not required for adaptation

**Figure 3:**
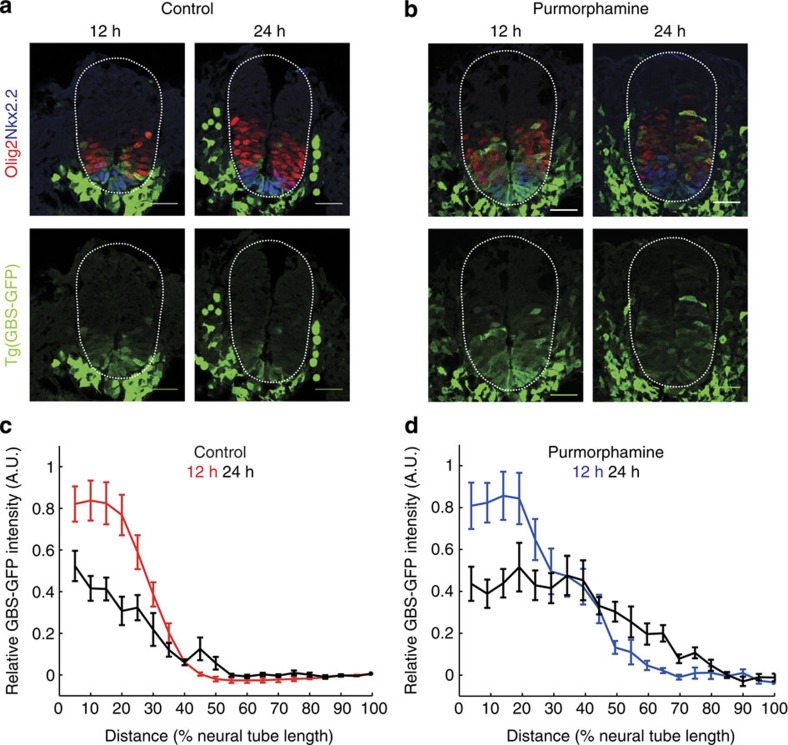
Ptch1 feedback is not required for adaptation. (**a**,**b**) Examples of mouse embryos at 4–6-somite stage cultured for 24 h in the presence (**b**) or absence (**a**) of Pur. The media was replaced after 12 h to ensure against degradation of Pur, and the expression of GBS-GFP was recorded at 12 and 24 h. The embryos were also stained for the neural progenitor transcription factors Nkx2.2 and Olig2. Scale bars, 20 μm. (**c**,**d**) The GBS-GFP fluorescence intensity quantified as in Fig. 1c. The plots show the average profiles from six experiments, normalized to the maximum GFP levels in each experiment at 12 h, separately for Control and Pur treatment. In both cases, there is an adaptation in the ventral region of the neural tube demonstrating that Ptch1 feedback is not required for adaptation.

The upregulation of Ptch1 has been suggested to explain adaptation^16^,^17^,^46^. To test whether Ptch1 feedback is sufficient to account for the adaptation, we took advantage of whole-embryo mouse culture and the small molecule Purmorphamine (Pur). This compound activates the pathway by direct interaction with Smo, thereby removing the effect of receptor feedback^47^. We cultured E8.5 GBS-GFP transgenic embryos in the presence or absence of 10 μM Pur, for 12 or 24 h (Fig. 3a,b, Methods). To ensure that Pur is not degraded during the time course of the experiment, we replaced the Pur-containing medium at 12 h. In control embryos, the GBS-GFP profiles showed the expected adaptation in the ventral neural tube between 12 and 24 h (Fig. 3c). Strikingly, the levels of GBS-GFP in the ventral neural tube of Pur cultured embryos also adapted, while in the dorsal neural tube, which had not been previously exposed to Shh, the levels of signalling increased (Fig. 3d). Thus, significant adaptation is retained even in the absence of Ptch1 mediated inhibition of signalling, consistent with the idea that Ptch1 feedback is not solely responsible for adaptation in this system.

### Alternative mechanisms can account for adaptation

**Figure 4:**
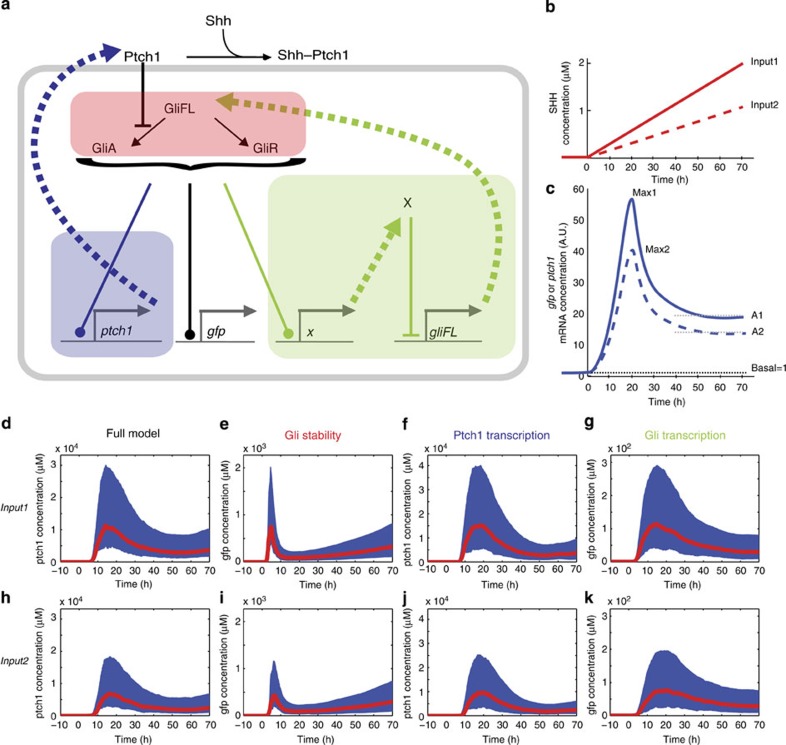
Three distinct mechanisms could explain adaptation. (**a**) Diagram of the full model used in the analysis. Shh binds to Ptch1, freeing the inhibition of GliFL conversion to GliA. GliA and GliR both compete to bind at two binding sites on the target genes gfp, ptch1 and *x*. *X* inhibits the production of GliFL. The coloured boxes highlight the three different mechanisms. Adaptation occurs by the regulation of Gli degradation (red) or adaptation by ptch1 feedback (blue) or by the transcriptional downregulation of Gli (green). (**b**,**c**) The experimental data (Fig. 2 and Supplementary Fig. 1c) was stereotyped into two time courses representing the average levels of Shh (**b**) and GBS-gfp and ptch1 (**c**) in two ventral regions of the neural tube (0–10% and 10–20% of relative DV length). The Shh time course (**b**) (from Fig. 2g) was linearly fitted and extrapolated back in time to zero levels. (**c**) The GBS-gfp and ptch1 target time dynamics were approximated from the protein expression in Fig. 2h,i. (**d**–**k**) Simulated signal trajectories of the posterior parameter distributions obtained by the Bayesian analysis. The Figures show the median trajectory (red) and 40–60 percentile ranges (blue) for either gfp (for the Gli stability and transcription models) or ptch1 (in the full model and ptch1 feedback model—where gfp followed similar trajectories). The response to the higher Shh input (**d**–**g**) and the lower Shh input (**h**–**k**) for the full model (**d**,**h**), Gli stability (**e**,**i**), Ptch1 transcription (**f**,**j**) and Gli transcription (**g**,**k**) are shown.

To explore mechanisms that could account for adaptation, we developed an Ordinary Differential Equation (ODE) model comprising key features of the Shh pathway (Fig. 4a, Supplementary Fig. 2a). The full model describes the binding of Shh to the receptor Ptch1 by mass action kinetics. For simplicity, we did not include a description of Smo, instead Ptch1 directly inhibits the conversion of GliFL into its activator form (GliA). In the absence of Shh, GliFL is converted into its repressor form (GliR). GliA and GliR both compete for binding at the same binding sites of target genes, which include *ptch1*, the *gfp* transgene and an intermediary transcription factor that we denote as ‘X’, which is potentially able to inhibit GliFL production. To account for any delays in the appearance of active Ptch1 protein that could result from translation and intracellular trafficking, *ptch1* mRNA was initially translated into an inactive protein before conversion to the active form that is able to bind to Shh.

Alongside the full model, we explicitly compared three models in which the different mechanisms functioned in isolation. First, we tested the regulation of Gli stability. We allowed the degradation rates of the different Gli isoforms to differ and correspondingly disabled the transcriptional regulation of Gli by transcription factor X and the regulation of Ptch1 by Gli (the transcription rates of *gli* and *ptch1* were made independent of X and Gli—Methods, Supplementary Fig. 2b). The second model represents only the negative feedback via Ptch1 upregulation. For this the transcriptional regulation of Gli by X was removed and the degradation rates of GliFL, GliA and GliR were fixed to be equal. The third model tests the transcriptional regulation of Gli, by fixing the degradation of rates of Gli to be equal and removing *ptch1* regulation by Gli.

As many of the parameters in the model are unknown, we used ABC^43^ performed with the software ABC-SysBio^48^,^49^ and cuda sim^50^ to analyse the model (Methods). This technique produces an approximate representation of the parameter space (the posterior distribution) for which a model is able to satisfy a particular target. In this case, we required that the dynamics of a transgenic reporter of Gli activity, as well as *ptch1*, show adaptation in response to an increasing concentration of Shh based on the data (Fig. 4b,c). To formulate this objectively, we used the data to generate the following idealized descriptors of Shh dynamics and signal adaptation. The Shh levels at two dorsoventral positions in the neural tube (0–10% and 10–20% of DV length) over time were fitted to linear temporal profiles and extrapolated back in time to zero (Fig. 4b). The basal levels of GBS-GFP induction (at time zero) were assumed to be equal to the level recorded far from the Shh source, at 50% of the dorsoventral tube size. The induction levels at the more ventral positions were determined relative to this basal level. In addition, we made the assumption that *gfp* and *ptch1* mRNA are induced to the same or higher relative levels as GFP protein within the same time interval.

The models were first simulated until a steady state in the expression of all proteins was reached (300 h) with the Shh concentration set to zero. Subsequently, the two increasing concentrations of Shh ligand were simulated over a 70-h period. In the Bayesian analysis, we specified a ‘distance function’ that selected for parameter sets that could satisfy the following requirements in the trajectory of *ptch1* and *gfp* mRNA: (1) Induction—the maximum level of signal induction (recorded within 40 h, relative to the basal level before Shh stimulation—denoted max1 and max2) was greater than 50-fold (for signal 1) or 30-fold (for signal 2); (2) Signal sensitivity—the response to the two different input signals was distinguishable, such that the relative level of maximum signal induction (max1/max2) was greater than 1.5; (3) Adaptation—the relative ratio of the max signal to the adapted level (the average signal between 40 and 70 h) in both signals was greater than 2.5. For the Gli stability regulation model and Gli transcriptional regulation model, only the trajectory of *gfp* mRNA was used in the distance function as in these models *ptch1* transcription was decoupled from the regulation of Gli proteins. We provided ABC-SysBio with a set of prior parameter ranges for each of the parameters (see Supplementary Fig. 2b). As many of the parameters in this model have not been determined, we used prior ranges spanning approximately four orders of magnitude (based on a review of equivalent biochemical rates).

**Figure 5:**
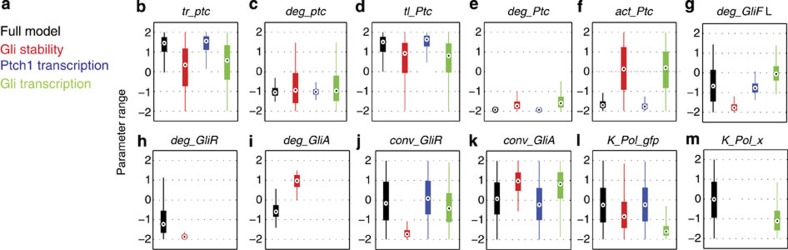
Posterior parameter distributions for adaptation mechanisms. (**a**–**m**) Box plots of the marginal posterior distributions for the key parameters for the indicated models. These comprise: the transcription rate of ptch1 (**b**), the degradation rate of ptch1 mRNA (**c**), the translation rate of ptch1 (**d**), the degradation rate of Ptch1 protein (**d**), the activation rate of Ptch1 (**f**), the degradation rates of GliFL (**g**), GliR (**h**), GliA (**i**), the conversion rates of GliFL to GliR (**j**) and GliFL to GliA (**k**), and the binding affinites of Polymerase to gfp (**l**) and to *x* (**m**).

For the full model and the three specific mechanisms, the Bayesian analysis was able to retrieve parameters that could fully satisfy the target distance function (all models show sufficient levels of induction, adaptation and sensitivity to Shh input levels that could qualitatively reproduce the experimental data). Figure 4d–k shows the distribution of the adapting signal trajectories for each of the models. Figure 5 compares the marginal posterior distributions obtained for the model parameters that were found to be most sensitive, plotted on the prior range. (The remaining parameters (Supplementary Fig. 2c) were not highly constrained in any of the models.)

Notably the Ptch1 transcriptional feedback and the full model have higher signal induction than the other two models (compare Fig. 4d,f,h,j with Fig. 4e,g,i,k). These models also require high transcription and translation of *ptch1* alongside stable mRNA and protein and slow rates of activation (Fig. 5). This suggests that, for most parameter regimes, the full model relies on Ptch1 feedback for adaptation. It also implies that Ptch1 feedback requires high levels of induction of Ptch1 to result in adaptation.

The Gli stability regulation model relies on low rates of GliFL degradation, GliR degradation and GliR conversion and a correspondingly high rate of GliA degradation and conversion (Fig. 5). As a result, this model has a relatively faster response time (Fig. 4e,i) and adapts quicker than the other models.

The Gli transcriptional regulation model is constrained only by requiring relatively low levels of polymerase binding for the Gli transcriptional targets, *gfp* and *x* (as exemplified by the low values for polymerase binding—*K_Pol_gfp* and *K_Pol_x* in Fig. 5). This can be attributed to negative feedback regulation of *gli* ensuring it is maintained at relatively low levels of expression. In order for low levels of Gli to be able to exact a response in the regulation of its targets, their corresponding levels of polymerase binding (and hence basal expression) must also be kept at similarly low levels. In summary, this analysis confirmed that each of the three mechanisms, acting alone or together in the combined model, could account for adaptation in the neural tube.

### Gli2 is downregulated by Shh signalling in the neural tube

**Figure 6:**
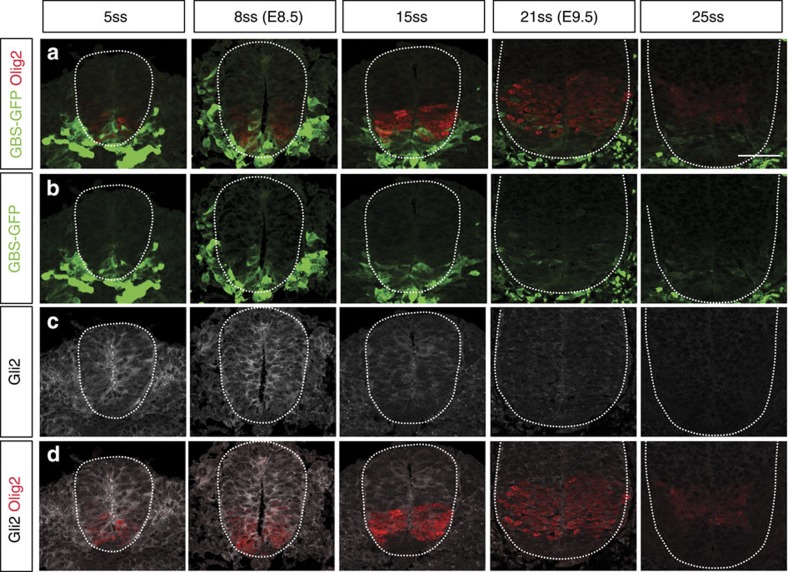
Gli2 is downregulated in the ventral neural tube. (**a**–**d**) Transverse sections of the neural tube between somite stages 5ss and 25ss were immunostained for GBS-GFP, Olig2 (a marker of the pMN domain), and Gli2. Gli2 protein levels are significantly downregulated by 15ss in the Nkx2.2 domain (ventral to Olig2) and across the entire neural tube by 25ss, when GBS-GFP expression has also been downregulated. Identical magnification in all panels; scale bar, 50 μm.

The computational screen suggested that the regulation of Gli stability and/or Gli production could contribute to the observed signalling dynamics and explain why adaptation is observed experimentally when Ptch1 regulation is bypassed (Fig. 3). In particular, a consequence of the Gli stability model is that sustained exposure to Shh ligand would cause a decrease of the total levels of Gli, because the GliFL pool is depleted and converted to the more unstable GliA isoform. Consistent with this, total Gli2 protein levels are reduced in embryos where high levels of Shh signalling are sustained by manipulating the signalling pathway upstream of Gli activation^34^,^51^. To further test whether Gli2 protein levels also decrease under physiological conditions, we assayed Gli2 levels by immunostaining at different stages in the neural tube (Fig. 6a–d). This revealed that the levels of Gli2 protein start decreasing at E9 (~15 somite stage), first ventrally to the Olig2 expression domain (in the p3 and putative floor plate domain), and then across the entire neural tube (Fig. 6c). Comparison with *gli2* mRNA (Supplementary Fig. 3—reproduced from ref. 18) suggests that transcription is also downregulated over time in ventral regions of the neural tube (see also refs 10, 14, 15). However, protein downregulation, particularly in more dorsal regions, appears to precede the disappearance of the mRNA (compare Fig. 6a–d with Supplementary Fig. 3). This suggests that the regulation of both transcription and protein stability could play a role in regulating the overall levels of Gli2 activity.

### Distinct Shh signalling dynamics in NIH3T3 fibroblasts

**Figure 7:**
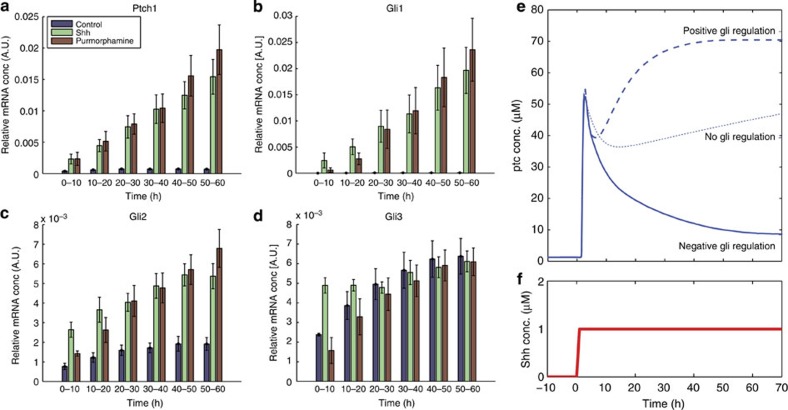
Different dynamics of Shh signalling in NIH3T3 cells. (**a**–**d**) NIH3T3 cells were cultured with Shh, Pur or control medium for the indicated amounts of time. Gli1, Gli2, Gli3 and Ptch1 RNA expression was determined by qPCR and normalized to actin levels. Signalling is induced as shown by the upregulation of Ptch1 and Gli1 however there was no adaptation. In these cells Gli2 and to a lesser extent Gli3 were upregulated by Shh signalling. (**e**,**f**) The model (described in Fig. 4a and Supplementary Fig. 2) was implemented with a fixed pulse of Shh (**f**). A set of parameters was selected for which adaptation would occur in ptch1 (and gfp—not shown; solid line in **e**). The parameter *c_X*, defining the transcriptional strength of factor *X*, was varied and the model output compared in each case (I): *c_X*=0 (X acts a repressor—solid line); *c_X*=1 (*X* has no effect—dotted line); *c_X*=10 (*X* acts as an activator—dashed line). The remaining model parameters were: *tl_Ptc*=100, *tl_X*=1, *tl_GliFL*=100, *conv_GliR*=0.01, *tr_gliFL*=100, *tr_x*=1, *deg_ptc*=2, *tr_ptc*=100, [Pol]=1, *deg_Ptc*=0.1, *deg_GliFL*=0.1, *deg_GliA*=1.5, *deg_GliR*=0.01, *deg_X*=0.5, *deg_x*=1, *K_Gli_ptc*=1, *deg_gliFL*=0.03, *conv_GliA*=10, *K_X_gli*=10, *K_Pol_ptc*=1, *K_Pol_x*=1, *K_Pol_gli*=0.01, *c_GliA*=10, *c_GliR*=0, *k_ShhPtc*=100, *Km_Ptch1*=1, *K_Gli_x*=10, *act_Ptc*=10, (units are defined in Supplementary Fig. 2B).

Previous studies indicated that, in contrast to neural progenitors, NIH3T3 cells do not display adaptation in response to Shh stimulation^42^. To confirm these observations, we exposed NIH3T3 cells to 16 nM Shh or 1 μM Pur for between 6 and 48 h. Samples were collected and gene expression analysed by quantitative PCR (qPCR; Fig. 7a–d, Methods). These data indicated that the levels of mRNA of the Shh-responding genes Gli1 and Ptch1 increased over the time course of the experiment, indicating a lack of adaptation in this cell type (Fig. 7a–d).

To test whether the signalling dynamics in NIH3T3 could be explained by a difference in the regulation of Gli production, we assayed expression of Gli2 and Gli3. In cells treated with Pur or Shh, there is significant upregulation of Gli2 expression (Fig. 7c). Gli3 is also upregulated, thus discounting the downregulation of Gli3 and its dominant repressor activity as an explanation for sustained signalling. Given that there is still significant upregulation of Ptch1 in this system, we conclude that the Gli2 regulation has a dominant influence on the overall signalling dynamics.

We took advantage of the *in silico* simulations to ask whether or not a change in Gli2 regulation could account for the distinct dynamics that we observed in NIH3T3 cells compared with the neural tube (Fig. 7e,f). A parameter set was selected with Gli transcription regulation and differential Gli stability in which *ptch1* expression adapted. To represent the NIH3T3 experiment, we stimulated the model with a pulse of Shh. We changed a single parameter in the model (*c_X*) that determines the transcriptional regulation of gliFL production by *X*. With *X* acting as a repressor (*c_X*=0), the signal strongly adapts; changing the parameter so that it is neutral (*c_X*=1) results in signal that continues to adapt (due to Ptch1 feedback and enhanced degradation of Gli) but the adaptation is less than with Gli transcriptional downregulation. Changing the parameters so that *X* acts as an activator (*c_X*=10) results in loss of adaptation, except for a short transient decrease due to the Ptch1 negative feedback. In this case, the expression of *ptch1* increases due to the transcriptional upregulation of Gli. This therefore supports the idea that differences in Gli2 regulation could explain the differences in signalling dynamics.

## Discussion

In this study, we have explored the dynamics of Shh signal transduction. The analysis indicates that Shh forms an exponential gradient of increasing amplitude in the neural tube. By contrast, the downstream Gli activity and Ptch1 levels initially increase but then adapt over a period of ~20 h. We investigated how specific connections between different components of the signalling pathway could account for adaptation in the signalling activity. We provide evidence that Ptch1 feedback is not the sole mechanism that accounts for adaptation. We show that Gli2 is downregulated in the neural tube and we attribute adaptation in the neural tube to Gli regulation either at the transcriptional level or by regulating the stability of the different protein isoforms. Consistent with this, the upregulation of Gli transcription in NIH3T3 fibroblasts provides an explanation for the sustained Shh signalling observed in these cells. Thus, the mechanism and structure of the signalling pathway provides sufficiently flexibility to allow signalling dynamics to be altered in different cell types.

In the ventral neural tube, where Shh controls the patterning of neural progenitors, we observed an exponential distribution of Shh protein. This gradient maintained an approximately constant decay length of ~20 μm over the development period in which Shh is required for patterning (Figs 1 and 2). This length scale corresponds to ~4 cell diameters and represents ~20% of the total dorsal–ventral length of the neural tube, at the earliest development time points (E8.5). In contrast to the constant decay length, the amplitude of the Shh gradient increases significantly during neural progenitor patterning. This increase in amplitude is likely to result from an increase in the number of cells expressing Shh as a result of the induction and proliferation of Shh-expressing floor-plate cells^17^,^18^. In addition, an increase in the level of expression of Shh within the floor plate might also contribute to increasing amplitude^13^. A consequence of the increasing amplitude and constant decay length is that, in contrast to other morphogens^3^, the Shh gradient does not scale with tissue size. Instead, ventral neural progenitors that are close to the source boundary are exposed to an increasing concentration of Shh, whereas more dorsally located progenitors are exposed to constant or even decreasing ligand levels. Accordingly, the temporal profiles are different at different DV positions in the neural tube. Moreover, the highest fold-induction of signalling activity corresponds to the position of the highest rates of Shh increase.

Negative feedback loops are well-established mechanisms for producing adaptation^26^,^27^ and the upregulation of Ptch1, as well as other inhibitors of Shh signalling, has previously been associated with adaptation in response to Shh^16^,^17^,^46^. However, the computational screen indicated that, faced with an increasing concentration of ligand, relying on Ptch1 feedback necessitates very high levels of Ptch1 upregulation to maintain free receptors to inhibit Smo activity and ensure low levels of signal. Moreover, experimentally activating Shh signalling by exposing mouse embryos to Pur, a small molecule that bypasses Ptch1 feedback by directly activating Smo, indicated that Ptch1 feedback was not sufficient to explain the adaptation (Fig. 3). Thus, adaptation continues in the absence of receptor level negative feedback. This suggests that one or more additional mechanisms must account for the observed signalling dynamics.

The Bayesian screen revealed that two alternative mechanisms (Gli transcriptional regulation and regulation of Gli protein degradation) could account for the signalling dynamics. We did not distinguish between Gli2 and Gli3 in this model. Notably signal adaptation is still present in Gli3^−/−^ mutant mice^14^, suggesting that the regulation of Gli3 is not sufficient to explain adaptation. Thus, the transcriptional or post-translation regulation of Gli2 appears to be crucial for the signalling dynamics in the neural tube. Taken together, the results of the computational screen can explain the experimental data from the Pur treatments (Fig. 3), the Gli2 expression profile (Fig. 6) and the distinct signalling dynamics in NIH3T3 cells (Fig. 7). Moreover, signal adaptation in response to Hedgehog signalling has also been reported in the *Drosophila* wing disc^52^ raising the possibility that similar mechanisms operate in this tissue.

In the Gli stability regulation model, the active form of Gli was significantly less stable than the inactive and repressor form. This is consistent with experimental data^30^,^31^,^32^,^33^,^34^. In this model, stable full-length Gli accumulates in the absence of Shh; in the presence of Shh, Gli is transformed into a more unstable activating form (that is, with a higher degradation rate). The increased rate of conversion from GliFL into the activated isoform on Shh exposure and the high degradation rate of the activated isoform lead to a stable decrease in the levels of GliFL and total Gli levels. This depletion of GliFL dampens the initially high signal. Thus, this system acts much like a capacitor discharging in an electric circuit. This type of transient response is common in other molecular systems that rely on integral control^19^,^36^,^37^,^38^,^39^,^40^.

The Gli transcriptional regulation model relies on activated Gli proteins inducing the expression of a transcription factor that inhibits Gli expression. It has previously been shown that Gli2 transcription is downregulated in the p3 domain^18^ where Nkx2.2 is expressed. Thus, Nkx2.2 could be a candidate for the regulation of *Gli2* transcription, similar to its role in regulating the expression of *Gli3* (ref. 53). Additional candidates, such as Nkx6.1 and Olig2, are also expressed in ventral neural progenitors in response to Shh signalling^11^. In this study, we showed that Gli2 protein levels were significantly downregulated across the whole neural tube at times when gli2 transcripts remain detectable dorsal to the p3 domain (Fig. 6a–d), suggesting that the regulation of stability is likely to contribute in addition to any transcriptional control.

The three types of adaptation mechanism described here are not mutually exclusive. Moreover, analogous mechanisms (of stability regulation and transcriptional regulation) could also operate via other pathway components such as Smo. While we do not rule this out, experimental findings are consistent with differential stability of active and inactive Gli proteins^30^,^31^,^32^,^33^,^34^ and the regulation of Gli transcription^18^,^53^.

The incorporation of multiple levels of regulative feedback into a morphogen patterned system has been dubbed General Relativistic Positional Information^54^ because it means there is no longer a simple biochemical correlate of position. Instead, the acquisition of cell identity becomes a function of the dynamic state of the system. What purpose would this serve in the neural tube? One possibility is that the adaptation ensures a limited time window for the transcriptional response to the morphogen. This would then allow the duration of signal to be used, in conjunction with the downstream transcriptional network, to generate distinct gene expression responses^14^,^55^. Moreover, adaptation in the response of cells, rather than downregulation of ligand production, enables the same morphogen to perform other regulatory functions in the tissue across a larger time span or spatial range. For example, after ventral pattern is established in the neural tube, Shh is required to direct commissural axon guidance from E10 (ref. 56) and to induce oligodendrocytes from E12.5 (refs 57, 58). Shh secreted from the notochord and floor plate is also used to control cell fate decisions in the scelerotome and dermomytome, adjacent to the dorsal neural tube, at later developmental times^59^,^60^. Adaptation means that the ventral neural progenitors become insensitive to further signalling and permits the Shh gradient to be reused for other purposes. In addition, it has been suggested that adapting signals arising from feed forward loops may provide a mechanism for reading the fold change in input signals^61^. Other studies have implicated rate of change detection as a way in which tissue growth may be controlled by morphogen gradients^62^.

Ptch1 feedback is likely to have a role that extends beyond assisting signal adaptation. Feedback is typically employed to ensure some robustness against variation in the properties of one of more system components. Receptor feedback, such as that displayed by Ptch1 in the neural tube, has been proposed to affect the shape and robustness of the ligand gradient by enhancing the degradation of the morphogen in a concentration-dependent manner^52^,^63^,^64^,^65^. Feedback through Ptch1 regulation may also play a role at the cellular level, perhaps helping to regulate against initial variation in receptor numbers^66^ or other pathway components.

By contrast to the neural tube, adaptation to Shh is not observed in NIH3T3 fibroblasts (Fig. 7)^42^. In these cells, as in the neural tube, Shh signalling results in a decrease in Gli3-repressor levels^30^. In addition, there was significant upregulation of the expression of Gli1, Gli2 and Gli3. Gli2 is the predominant activating form of Gli in amniotes^67^ and its upregulation could provide the mechanism to explain the non-adapting dynamics. Consistent with this, incorporating upregulation of Gli into the computational simulations demonstrated that this was sufficient to counter the adaptation that would occur due to Gli processing and Ptch1 feedback. Thus, increasing the flux of total Gli into the system allows a higher steady state to be maintained following ligand activation and this can compensate for the increased degradation of the activated Gli. This exemplifies how a relatively subtle change in the signalling network can produce qualitative changes in the behaviour of the system. This flexibility may be an important feature of the Shh pathway that has allowed it to be modified for different purposes in different cell types in a range of tissues. This flexibility is likely to be found in other signalling pathways and help explain how a relatively small number of signalling pathways are reused multiple times during tissue development and homeostasis.

## Methods

### Mouse strains

*Tg(GBS-GFP)* reporter transgenic mice contain a Gli reporter consisting of concatemerized GBS upstream of the hsp68 minimal promoter and eGFP^14^. All procedures performed on mice in this study were carried out according to the United Kingdom Home Office regulations under the project license PPL80/2528 and approved by the Animal Welfare and Ethical Review Panel of the MRC-National Institute for Medical Research.

### Staging of embryo sections

Embryos were staged by counting the number of somites. Subsequently, the brachial region (somites ~6–10) was taken for cryosectioning and immunohistochemistry. Several sections were analysed from each embryo. To produce a more objective measure of developmental age, we have staged each section by its total DV neural tube length, measured from the ventral to dorsal midline. Dorsoventral length correlated linearly with somite stage (see Supplementary Fig. 1a).

### Immunohistochemistry

For the time-course characterization of GBS-GFP, Ptch1 and Shh, 68 embryos representative of somite stages from E8.5 to E10.5 were collected and several sections were analysed from the brachial region of each embryo. A subset of the sections was co-stained for GFP, Ptch1 and Nkx2.2; a second subset was stained for GFP, Shh and Nkx2.2 and a third subset for Shh, Nkx2.2 and Olig2. The staining for GFP, Nkx2.2 and Olig2 was previously published^14^ and the same data were reanalysed in this work. In total, the data comprise of *n*=184 Shh, *n*=206 GFP and *n*=102 Ptch1 sections. Primary antibodies used were mouse anti-Shh (1:10, Developmental Studies Hybridoma Bank, 5E1), guinea pig anti-Gli2 (ref. 68; 1:800, gift from Johnathan Eggenschwiller). The immunostaining was performed simultaneously on all sections from the time course.

### Quantification of protein levels

The immunostained sections from the whole time course were imaged using a Leica SP5 confocal system. Imaging was performed using the same settings, on the same microscope in the same day to minimize technical variability. Each image was the average of three optical sections, 0.2 μm apart, taken from the middle of a 14-mm cryosection. Fluorescence intensity was measured in rectangles of 16 μm wide positioned from the ventral to dorsal midline along the apical side of the neural tube. Image J v.1.43 g image analysis software (NIH) was used. Background measurements were taken from mesoderm subtracted from each assayed profile^14^. The data were binned (Fig. 2) across different developmental stages (as determined by embryo size—each bin represented 40 μm of increased DV length) and positions (extending across 10% of the DV length). The 95% confidence interval in the mean was determined for each temporal and spatial bin. The curves were smoothed by interpolation using the Matlab ‘Spline’ method with *x* intervals of 0.1 μm.

### Mouse embryo culture

Mouse embryos from 8 to 12 h.p.h. stages with intact yolk sacs were cultured for 12 or 24 h in media containing rat serum, Tyrode solution (1:1). Cultures were performed in a water-saturated roller-tube incubator at 37 °C, 5% CO_2_ and 20% O_2_. After culture, the embryos were fixed and processed^14^. Embryos expressing *Tg(GBS-GFP)* at the three-somite stage were used in the experiments. Pur (Calbiochem) was dissolved in dimethylsulphoxide and used at a concentration of 10 μM and for the 24-h time point, the Pur-containing medium was replaced at 12 h to ensure that Pur degradation during the culture time is not a factor affecting the result. The quantifications resulted from four independent experiments, with one to three embryos per condition analysed in each experiment.

### NIH3T3 culture

NIH3T3 mouse embryonic fibroblasts (obtained from the American Type Culture Collection) were cultured in DMEM containing 4.5 g l^−1^ D-Glucose and 0.11 g l^−1^ sodium pyruvate, supplemented with 2 mM L-glutamine, 100 U ml^−1^ Penicillin, 100 μg ml^−1^ Streptomycin (all Gibco) and 10% new-born calf serum (MP Biomedicals). For assays, the cells were seeded in four-well plates, grown to confluence, then cultured for between 6 and 40 h in 0.5% serum in the presence of bacterially produced Shh-N (16 nM)^69^, Pur (1 μm; Calbiochem) or left unstimulated. Fresh ligand-containing medium was added after 24 h of culture. Cells were harvested in Trizol and complementary DNA was synthesized from RNA with Superscript 3 (Life Technologies). qPCR was performed using an AB 7900HT qPCR machine and Platinum SYBR green Supermix (Life Technologies). Primers used were: Gli1–F-5′TTATGGAGCAGCCAGAGAGA,R-GAGCCCGCTTCTTTGTTAAT-3′; Gli2–F-5′TGAAGGATTCCTGCTCGTG-3′, R-5′-GAAGTTTTCCAGGACAGAACCA-3′; Gli3–F-5′-AAGCGGTCCAAGATCAAGC,RTGTTCCTTCCGGCTGTTC-3′; Ptc1–F-5′-TGACAAAGCCGACTACATGC,R-AGCGTACTCGATGGGCTCT-3′; Actin–F-5′-TGGCTCCTAGCACCATGA,R-CCACCGATCCACACAGAG-3′.

### Bayesian model analysis

To explore the different mechanisms that could account for adaptation, we developed a model comprising the key features of the Shh pathway described by system of ODEs (see Fig. 4a and Supplementary Fig. 2a). The full model describes the binding of Shh to the receptor Ptch1 (denoted ‘Ptc’ in equations) by mass action binding. Ptch1 directly inhibits the conversion of GliFL into its activator form (GliA)—this is decribed through a Hill function with a maximum conversion rate *conv_GliA*. In the absence of Ptch1, GliFL is converted into its repressor form (GliR) at a conversion rate, *conv_GliR*. GliA and GliR both compete to bind at the same binding sites of target genes which include ptch1, the GFP reporter and an intermediary transcription factor that we denote as ‘*x*’ which is potentially able to inhibit GliFL production.

We use a thermodynamic function to describe the gene regulation in this system^70^ that could account for binding of different transcription factors at multiple binding sites. This description of gene regulation assumes that polymerase binding at a genes promoter will be modulated by the concentration of bound transcription factors. The strength of transcriptional activation is determined by a cooperativity factor c greater than 1. The inhibition by a repressor is assumed to be strong with *c*=0 for all repressor genes (*c_GliR* is not shown in the equations as this was always assumed to act as a repressor). Degradation rates are denoted *deg_*, transcription rates *tr_*, translation rates *tl_*, protein conversion rates *conv_*, protein activation rates *act_*, forward reaction rates *k_* and binding affinities *K_*.

To account for any potential delays in the upregulation of Ptch1 that could help facilitate adaptation, ptch1 RNA was initially translated into an inactive form (denoted Ptc_inactive) before conversion to the active form (denoted Ptc) at a rate *act_Ptc*. To compare the three different mechanisms that could account for adaptation, we modified the model in each case to disable the alternative mechanisms. In the first model where only enhanced Gli degradation can occur, the transcription was set to a constant value independent of Gli regulation and the transcription factor X was removed from the model so that Gli transcription was also set to a constant. Here the constant rates were determined solely by the rates of polymerase binding to the enhancers. In the second model where only Ptch1 upregulation can occur, the regulation of Gli by X was also disabled and the degradation rates of GliFL, GliA and GliR were fixed to be equal. In the final model, which relies on the transcriptional regulation of Gli, the degradation of rates of Gli was fixed and Ptch1 regulation by Gli was disabled.

We used ABC^43^ performed with the software ABC-SysBio^48^,^49^ and cuda-sim^50^ to analyse our model. This technique can be used to determine an approximate representation of the parameter space for which a model is able to satisfy a particular target. We stereotyped the data (from Fig. 2) in the way illustrated in Fig. 4b,c. The Shh time course for two relative positions in the tube (0–10% and 10–20%) was fitted to linear trajectory extrapolated back in time to zero (Fig. 4b) and normalized so they had a maximum concentration of either 2 or 1 respectively, In the analysis software, the ODE models were first simulated for long enough to reach a steady state in the expression of all proteins (300 h) and then stimulated with each of two increasing Shh signals over a 70-h period.

In the Bayesian analysis, we specified a ‘distance function’ that selected for parameter sets that could satisfy the following requirements in the trajectory of ptch1 and gfp RNA: (1) the maximum level of signal induction (recorded within 40 h, relative to the basal level before Shh stimulation—denoted max1 and max2) was greater than 50-fold (for signal 1) or 30-fold (for signal 2); (2) the relative level of signal induction (max1/max2) was greater than 1.5; (3) the relative ratio of the max signal to the adapted level (the average signal between 40 and 70 h) in both signals was greater than 2.5. Note that for the Gli degradation model and Gli regulation model only the trajectory of gfp RNA was used in the distance function as in these models ptch1 transcription was artificially detached from the regulation of Gli proteins.

The distance function used an incremental scoring system that was minimized as each of the ratios approached their minimum value (that is, for the adaptation ratio; <1: score 10, <1.1: score 5, <1.5: score 2, <2: score 1, ≥2.5: score 0)—in all cases, all the models were able to fully satisfy the minimum possible score of zero. Induction and adaptation ratios were allowed to exceed the minimum required values without affecting the score. We also added into the distance function the requirement that GliA would respond and adapt (implicit in all the mechanisms) as this enabled a more efficient search of the parameter space. We provided ABC-SysBio with uniform prior ranges on a log scale for each of the parameters (see Supplementary Fig. 2b). As many of the parameters in this model have not been determined, we used prior ranges spanning approximately four orders of magnitude based on a literature search of equivalent biochemical rates.

The inference was run with 1,000 particles (parameter sets), which are simulated by the ODE model. ABC-SysBio uses an SMC algorithm to efficiently search the parameter space for those that can satisfy the model target^43^. The output is a set of 1,000 weighted parameter sets that represent the posterior distribution of parameters. The software run time was ~1 week with each model running on a separate GPU. In Fig. 7e,f, a parameter set obtained from the Gli regulation model was adapted to model the NIH3T3 experiment—ptch1 regulation by Gli was reinstated so that ptch1 expression could adapt. The parameter *c_X* was varied to contrast the different effects of Gli regulation on ptch1 (and other target gene) dynamics.

## Author contributions

M.C. conceived the project, performed data analysis, computational modelling and wrote the manuscript. A.K. performed the embryo culture experiment and Gli2 immunostaining, contributed to data analysis and wrote the manuscript. A.R. measured the Shh, Ptch1, GBS-GFP and Nkx2.2 profiles. R.B. performed the cell culture experiments. K.M.P. performed computational modelling and provided technical advice. C.P.B. performed computational modelling and provided technical advice. J.B. conceived the project and wrote the manuscript.

## Additional information

**How to cite this article:** Cohen, M. *et al.* Ptch1 and Gli regulate Shh signalling dynamics via multiple mechanisms. *Nat. Commun.* 6:6709 doi: 10.1038/ncomms7709 (2015).

## Supplementary Material

Supplementary Information Supplementary Figures 1-3 and Supplementary References
