# Supplementary material for: Ptch1 and Gli regulate Shh signalling dynamics via multiple mechanisms
Source: Nat Commun. 2015 Apr 2;6:6709. doi: 10.1038/ncomms7709 (PMC4396374; doi:10.1038/ncomms7709)
Supplement: Supplementary Information — Supplementary Figures 1-3 and Supplementary References [file ncomms7709-s1.pdf]

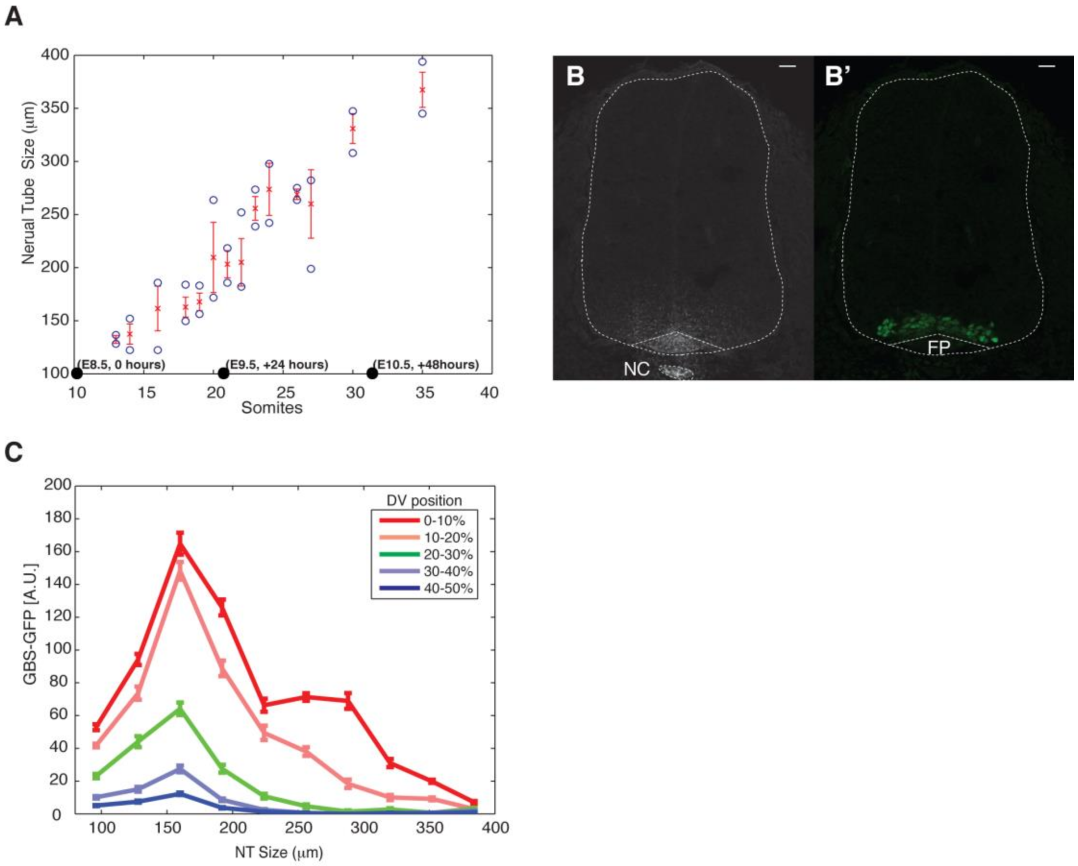

<u>Supplementary Figure 1</u> **Comparing Shh with GBS-GFP at different developmental times reveals a non-linear relationship.**

A To provide more accurate staging of neural tube development we exploited the linear relationship between somite number and neural tube size after E9.5 of development (along the ventral-dorsal axis). The mean is indicated by the red cross, the standard deviation by the error bar and the max and min by the blue circles. Each somite represents approximately 2 hours of developmental time, hence somite stage and embryonic day staging are related as labeled on the x-axis.

B A section from an embryo at the 30 somite stage immunostained for Shh (C) and Nkx2.2 (C'). The ventral limit of Nkx2.2 expression at this stage corresponds to the floor plate boundary as shown in Supplementary Figure 1B.

C An independent data set was derived for the GBS-GFP. The data were collected from 23 differently staged embryos and comprises 65 sections. The plot was derived in the same way as in Figure 2 (in this case there is no interpolation of the curves and error bars). The figure reveals the same temporal adaptation over time for each different position of the neural tube (data has been reproduced from ref. 1).

A

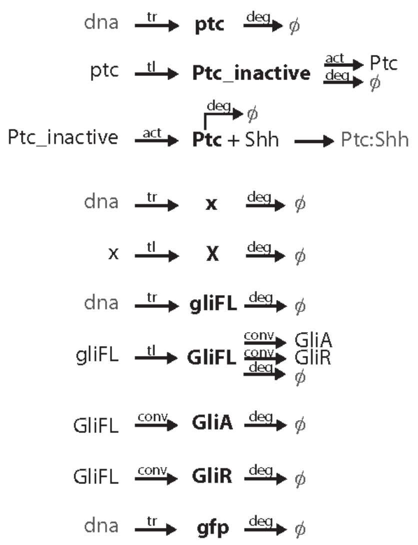

$$\frac{d([ptc])}{dt} = +\left(\frac{tr\_ptc \cdot (K\_Gli\_ptc \cdot [GliA] \cdot c\_GliA + 1)^2 \cdot K\_Pol\_ptc \cdot [Pol]}{K\_Pol\_ptc \cdot [Pol] \cdot (K\_Gli\_ptc \cdot [GliA] \cdot c\_GliA + 1)^2 + (K\_Gli\_ptc \cdot [GliA])^2 + (K\_Gli\_ptc \cdot [GliR])^2 + 2 \cdot K\_Gli\_ptc \cdot [GliR] + 2 \cdot K\_Gli\_ptc \cdot [GliA] + 2 \cdot K\_Gli\_ptc \cdot [GliR] \cdot K\_Gli\_ptc \cdot [GliA] + 1}\right) - (deg\_ptc \cdot [ptc])$$

$$\frac{d([PtcInactive])}{dt} = +(tl\_Ptc \cdot [ptc]) - (deg\_Ptc \cdot [PtcInactive]) - (act\_Ptc \cdot [PtcInactive])$$

$$\frac{d([Ptc])}{dt} = +(act\_Ptc \cdot [PtcInactive]) - (deg\_Ptc \cdot [Ptc]) - (k\_ShhPtc \cdot [Ptc] \cdot [Shh])$$

$$\frac{d([x])}{dt} = +\left(\frac{tr\_x \cdot (K\_Gli\_x \cdot [GliA] \cdot c\_GliA + 1)^2 \cdot K\_Pol\_x \cdot [Pol]}{K\_Pol\_x \cdot [Pol] \cdot (K\_Gli\_x \cdot [GliA] \cdot c\_GliA + 1)^2 + (K\_Gli\_x \cdot [GliA])^2 + (K\_Gli\_x \cdot [GliR])^2 + 2 \cdot K\_Gli\_x \cdot [GliR] + 2 \cdot K\_Gli\_x \cdot [GliA] + 2 \cdot K\_Gli\_x \cdot [GliR] \cdot K\_Gli\_x \cdot [GliA] + 1}\right) - (deg\_x \cdot [x])$$

$$\frac{d([X])}{dt} = +(tl\_X \cdot [x]) - (deg\_X \cdot [X])$$

$$\frac{d([gliFL])}{dt} = +\left(\frac{tr\_gliFL \cdot K\_Pol\_gli \cdot [Pol] \cdot (1 + K\_X\_gli \cdot [X] \cdot c\_X)^2}{(1 + K\_X\_gli \cdot [X])^2 + K\_Pol\_gli \cdot [Pol] \cdot (1 + K\_X\_gli \cdot [X] \cdot c\_X)^2}\right) - (deg\_gliFL \cdot [gliFL])$$

$$\frac{d([GliFL])}{dt} = +(tl\_GliFL \cdot [gliFL]) - \left(\frac{conv\_GliA \cdot [GliFL] \cdot Km\_Ptc}{Km\_Ptc + [Ptc]} + conv\_GliR \cdot [GliFL]\right) - (deg\_GliFL \cdot [GliFL])$$

$$\frac{d([GliA])}{dt} = +\left(\frac{conv\_GliA \cdot [GliFL] \cdot Km\_Ptc}{Km\_Ptc + [Ptc]}\right) - (deg\_GliA \cdot [GliA])$$

$$\frac{d([GliR])}{dt} = +(conv\_GliR \cdot [GliFL]) - (deg\_GliR \cdot [GliR])$$

$$\frac{d([gfp])}{dt} = +\left(\frac{tr\_gfp \cdot (K\_Gli\_gfp \cdot [GliA] \cdot c\_GliA + 1)^2 \cdot K\_Pol\_gfp \cdot [Pol]}{K\_Pol\_gfp \cdot [Pol] \cdot (K\_Gli\_gfp \cdot [GliA] \cdot c\_GliA + 1)^2 + (K\_Gli\_gfp \cdot [GliA])^2 + (K\_Gli\_gfp \cdot [GliR])^2 + 2 \cdot K\_Gli\_gfp \cdot [GliR] + 2 \cdot K\_Gli\_gfp \cdot [GliA] + 2 \cdot K\_Gli\_gfp \cdot [GliR] \cdot K\_Gli\_gfp \cdot [GliA] + 1}\right) - (deg\_gfp \cdot [gfp])$$

B

Parameter	Units	Description	Uniform Prior Range (Log10 scale, null = zero value)			
			Full Model	*Gli Degradation*	*Gli Regulation*	*Ptc Regulation*
**deg_GliFL**	h^{-1}	degradation rate of ful-length Gli protein	-2 : 1.5	-2 : 1.5	-2 : 1.5	-2 : 1.5
**deg_GliR**	h^{-1}	degradation rate of repressor Gli protein	-2 : 1.5	-2 : 1.5	**=degGliFL**	**=degGliFL**
**deg_GliA**	h^{-1}	degradation rate of active Gli protein	-2 : 1.5	-2 : 1.5	**=degGliFL**	**=degGliFL**
**deg_Ptc**	h^{-1}	degradation rate of Ptch1 protein	-2 : 1.5	-2 : 1.5	-2 : 1.5	-2 : 1.5
**deg_X**	h^{-1}	degradation rate of transcription factor 'X' protein	-2 : 1.5	**null**	-2 : 1.5	**null**
**deg_gfp**	h^{-1}	degradation rate of gfp RNA	-2 : 1.5	-2 : 1.5	-2 : 1.5	-2 : 1.5
**deg_gliFL**	h^{-1}	degradation rate of ful-length gli RNA	-2 : 1.5	-2 : 1.5	-2 : 1.5	-2 : 1.5
**deg_ptc**	h^{-1}	degradation rate of ptch1 RNA	-2 : 1.5	-2 : 1.5	-2 : 1.5	-2 : 1.5
**deg_x**	h^{-1}	degradation rate of transcription factor 'x' RNA	-2 : 1.5	**null**	-2 : 1.5	**null**
**k_ShhPtc**	h^{-1}	forward reaction rate of Shh Ptch1 binding	-2 : 2	-2 : 2	-2 : 2	-2 : 2
**K_Gli_gfp**	μM^{-1}	binding affinity of Gli to gfp enhancer	-2 : 2	-2 : 2	-2 : 2	-2 : 2
**K_Gli_ptc**	μM^{-1}	binding affinity of Gli to ptch1 enhancer	-2 : 2	**null**	**null**	-2 : 2
**K_Gli_x**	μM^{-1}	binding affinity of Gli to x enhancer	-2 : 2	-2 : 2	-2 : 2	-2 : 2
**K_X_gli**	μM^{-1}	binding affinity of X to gli enhancer	-2 : 2	**null**	-2 : 2	**null**
**K_Pol_gfp**	μM^{-1}	binding affinity of Polymerase to gfp promoter	-2 : 2	-2 : 2	-2 : 2	-2 : 2
**K_Pol_gli**	μM^{-1}	binding affinity of Polymerase to gli promoter	-2 : 2	-2 : 2	-2 : 2	-2 : 2
**K_Pol_ptc**	μM^{-1}	binding affinity of Polymerase to ptch1 promoter	-2 : 2	-2 : 2	-2 : 2	-2 : 2
**K_Pol_x**	μM^{-1}	binding affinity of Polymerase to x promoter	-2 : 2	**null**	-2 : 2	**null**
**Km_Ptc**	μM	Michaelis constant for Ptch1 inhibtion of Gli conversion	-2 : 2	-2 : 2	-2 : 2	-2 : 2
**Pol**	μM	background concentration of Polymerase	0	0	0	0
**tl_GliFL**	h^{-1}	translation rate of Gli (into full length form)	-2 : 2	-2 : 2	-2 : 2	-2 : 2
**tl_Ptc**	h^{-1}	translation rate of Ptch1 (into inactive form)	-2 : 2	-2 : 2	-2 : 2	-2 : 2
**tl_X**	h^{-1}	translation rate of X	-2 : 2	**null**	-2 : 2	**null**
**tr_gfp**	$\mu M h^{-1}$	maximal transcription rate of gfp	-2 : 2	-2 : 2	-2 : 2	-2 : 2
**tr_gliFL**	μMh-1	maximal transcription rate of gli	-2 : 2	-2 : 2	-2 : 2	-2 : 2
**tr_ptc**	μMh-1	maximal transcription rate of ptch1	-2 : 2	-2 : 2	-2 : 2	-2 : 2
**tr_x**	μMh-1	maximal transcription rate of x	-2 : 2	**null**	-2 : 2	**null**
**act_Ptc**	h^{-1}	activation rate of Ptch1 (or transport rate to membrane)	-2 : 2	-2 : 2	-2 : 2	-2 : 2
**conv_GliA**	h^{-1}	conversion rate of GliFL to GliA	-2 : 2	-2 : 2	-2 : 2	-2 : 2
**conv_GliR**	h^{-1}	conversion rate of GliFL to GliR	-2 : 2	-2 : 2	-2 : 2	-2 : 2
**c_GliA**		transcriptional power of GliA	1	1	1	1
**c_X**		transcriptional power of X	**null**	**null**	**null**	**null**

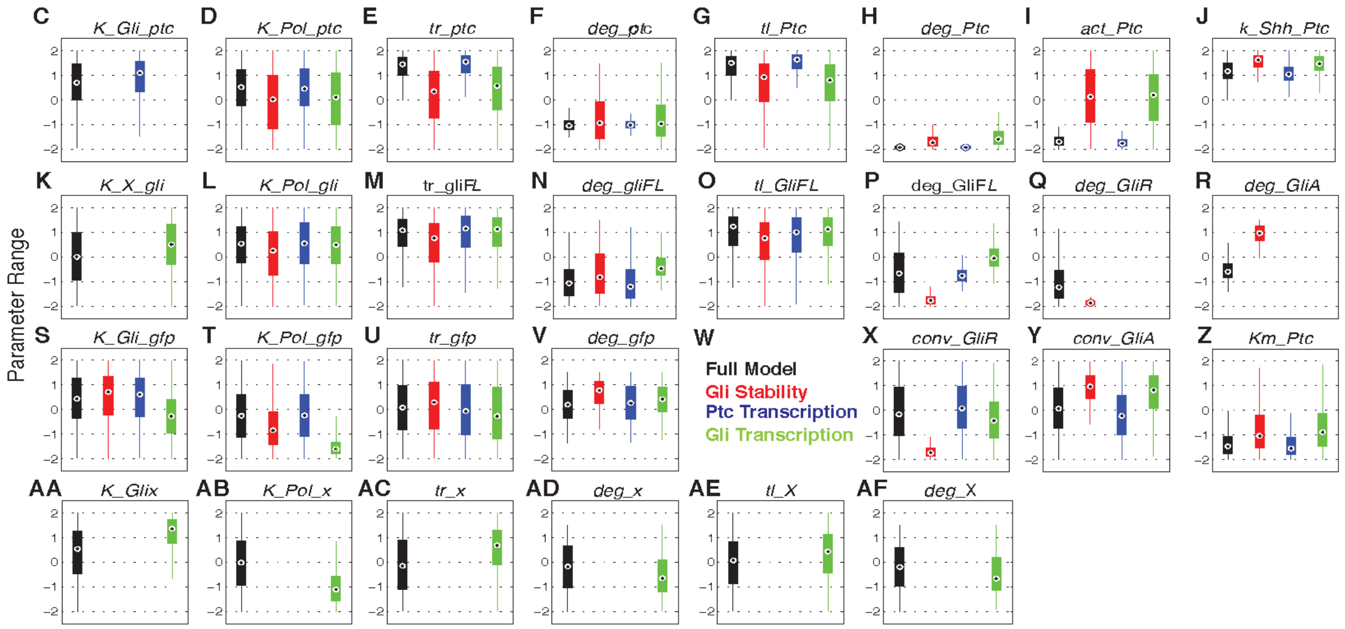

<u>Supplementary Figure 2</u> Bayesian model analysis and posterior parameter distributions.

A The full set of reactions used in the model. The reaction schema for each protein or RNA are illustrated (left) alongside a set of ordinary differential equations (ODE)s (right) which detail the dynamics and regulatory functions associated with each reaction. Degradation rates are denoted *deg_*, transcription rates *tr_*, translation rates *tl_*, protein conversion rates *conv_*, protein activation rates *act_*, forward reaction rates *k_*, and binding affinities *K_*. Shh binds to Ptch1 (abbreviated to Ptc) by mass action binding. Ptch1 inhibits GliFL conversion to GliA via a first order Hill function. Thermodynamic regulation functions are used to determine gene expression in all cases – binding of all the transcription factors is assumed to occur at two independent sites. (Note that GliR is assumed to always act as a strong repressor with *c_GliR*=0 hence these terms are not shown in the regulatory function).

B The full set of parameters used in the model and their associated units. The prior ranges used in the Bayesian analysis are shown for each of the model mechanisms. Log uniform ranges were used in all cases - hence a value of 0 indicates 1, where a zero value was used in the model this is indicated by the *null* value in the table. When the Gli degradation mechanism was disabled the degradation rates of GliA and GliR were set to equal the degradation rate of GliFL.

C-AF The marginal posterior distribution is shown for each of the derived parameters in the different models, coloured according to the key in W. The box plots show the median (central bar), 25-75 percentiles (box) and ~1-99 percentiles (whiskers) of the marginal distribution for each of the parameters indicated. Note that in the cases where no boxplot is shown this indicates that the parameter did not feature in that particular mechanism (i.e was fixed at zero). The parameters are described in full in Supplementary Figure 2B where the prior ranges and units are specified.

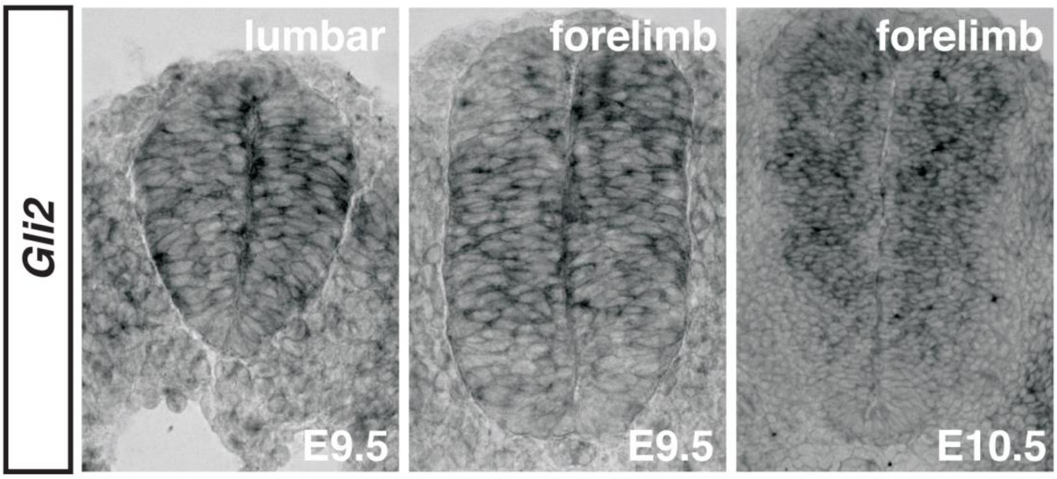

Supplementary Figure 3 **Gli2 transcriptional regulation in the neural tube**

This figure is reproduced from ref. 2. G-J In situ staining of Gli2 transcripts reveals that Gli2 transcriptional down regulation between E9.5 and E10.5 is restricted to ventral regions of the neural tube.

REFERENCES

1. Balaskas, N. *et al.* Gene regulatory logic for reading the Sonic Hedgehog signaling gradient in the vertebrate neural tube. *Cell* **148,** 273–284 (2012).
2. Ribes, V. *et al.* Distinct Sonic Hedgehog signaling dynamics specify floor plate and ventral neuronal progenitors in the vertebrate neural tube. *Genes Dev.* **24,** 1186–1200 (2010).
